# Epidemiology of Community-Onset *Staphylococcus aureus* Bacteremia

**DOI:** 10.5811/westjem.2019.2.41939

**Published:** 2019-04-16

**Authors:** James Y. Yarovoy, Andrew A. Monte, Bryan C. Knepper, Heather L. Young

**Affiliations:** * Upstate Medical University, Department of Emergency Medicine, Syracuse, New York; † University of Colorado, Department of Emergency Medicine, Denver, Colorado; ‡ Denver Health Medical Center, Department of Patient Safety and Quality, Denver, Colorado; § Denver Health Medical Center, University of Colorado, Department of Medicine, Denver, Colorado

## Abstract

**Introduction:**

*Staphylococcus aureus* bacteremia (SAB) is the second-most common cause of community-onset (CO) bacteremia. The incidence of methicillin-resistant *S. aureus* (MRSA) has recently decreased across much of the United States, and we seek to describe risk factors for CO-MRSA bacteremia, which will aid emergency providers in their choice of empiric antibiotics.

**Methods:**

This is a retrospective cohort study of all patients with SAB at a 500-bed safety net hospital. The proportion of S. aureus isolates that were MRSA ranged from 32–35% during the study period. Variables of interest included age, comorbid medical conditions, microbiology results, antibiotic administration, duration of bacteremia, duration of hospital admission, suspected source of SAB, and Elixhauser comorbidity score. The primary outcome was to determine risk factors for CO-MRSA bacteremia as compared to methicillin-susceptible *S. aureus* (MSSA) bacteremia in patients admitted to the hospital through the emergency department.

**Results:**

We identified 135 consecutive patients with CO-SAB. In comparison to those with MSSA bacteremia, patients with MRSA bacteremia were younger (odds ratio [OR] 0.5, 95% confidence interval [CI], 0.4–0.7) with higher Elixhauser comorbidity scores (OR 1.4, 95% CI, 1.1–1.7). Additionally, these patients were more likely to have a history of MRSA infection or colonization (OR 8.9, 95% CI, 2.7–29.7) and intravenous drug use (OR 2.4, 95% CI, 1.0–5.7).

**Conclusion:**

SAB continues to be prevalent in our urban community with CO-MRSA accounting for almost one-third of SAB cases. Previous MRSA colonization was the strongest risk factor for current MRSA infection in this cohort of patients with CO-SAB.

## INTRODUCTION

*Staphylococcus aureus* bacteremia (SAB) is the second-most common cause of community-onset (CO) bacteremia, affecting 15–40 per 100,000 population per year.^1^,^2^ It is associated with a 20% mortality rate^3^,^4^ with higher mortality linked to the presence of methicillin resistance, comorbid conditions, intensive care unit admission, and prior exposure to antibiotics.^5^ Three-quarters of SAB are CO bacteremia, with the majority being secondary to skin and soft tissue infections. While the incidence of hospital-acquired SAB is decreasing, the incidence of CO-SAB has remained stable.^6^

The epidemiology of methicillin-resistant *S. aureus* (MRSA) has changed over the past four decades. Initially, MRSA was identified as a healthcare-associated pathogen.^7^ In the late 1980s, community-onset MRSA, primarily the USA300 strain, was first identified. It spread throughout healthy community members including children, athletes, military personnel, and inmates in the 1990s.^8^,^9^ By the mid-2000s, the USA300 strain became the predominant strain of MRSA in both CO and healthcare-associated cases.^10^ In fact, MRSA accounted for approximately 50% of all *S. aureus* cases at its peak. Most recently, MRSA has been decreasing in comparison to methicillin-susceptible *S. aureus* (MSSA).^6^

Risk factors for CO-MRSA have been poorly described in the recent era of decreasing MRSA prevalence. An evaluation of current risk factors for CO-MRSA is important for emergency medicine (EM) providers because it can impact the choice of empiric antibiotic therapy and prompt the early initiation of infection control measures. The goal of this study was to describe risk factors for CO-MRSA in a cohort of outpatients presenting to the emergency department (ED) with CO-SAB.

## METHODS

This is a retrospective cohort study of all patients with SAB at a 500-bed safety net hospital. The proportion of *S. aureus* isolates that were MRSA ranged from 32–35% during the study period. Patients were identified by review of the microbiology blood culture log. We included consecutive patients ≥ 18 years old with SAB occurring before hospital day three between June 1, 2013, and April 30, 2015, admitted through the ED. Pediatric patients, those with subsequent episodes of SAB during the study period, and those with incomplete microbiology data were excluded. Clinical and microbiological data were collected by manual review of the electronic medical record.

The primary outcome was to determine risk factors for CO-MRSA bacteremia, as compared to MSSA bacteremia. Variables of interest were predetermined before the study began and included age, comorbid medical conditions, presence of indwelling medical devices including orthopedic hardware and intravascular devices (i.e., pacemakers, prosthetic heart valves, arterial grafts, and patches), microbiology results, antibiotic administration, duration of bacteremia, duration of hospital admission, suspected source of SAB, and Elixhauser comorbidity score.^11^ The Elixhauser comorbidity score is a collection of 30 variables that are predictive of inhospital mortality.

The infectious diseases service performs a consultation on all patients with SAB. The suspected source of SAB was determined by an infectious diseases specialist (Heather L. Young) reviewing the infectious diseases consultation notes and using the following guidance to define the source of SAB:

- Skin and soft tissue infection: cellulitis or purulence in the superficial skin layers without a deeper underlying source and without a history of injection drug use (IDU).
- Vascular access: (1) pain, erythema, or phlebitis at a recent or current peripheral intravenous (IV) catheter, at a recent phlebotomy site, or overlying an arteriovenous fistula; or (2) a central venous catheter, including hemodialysis catheter, with pain, erythema, or purulence at the insertion site or without those symptoms but with no other recognized source of infection.^12^
- Bone or joint infection: purulence or a positive culture for *S. aureus* isolated from bone or synovial fluid.
- IDU: skin and soft tissue infection at a site used for injecting drugs.
- Pneumonia: pulmonary infiltrates accompanied by hypoxia.
- Urinary tract infection: a urine culture positive for *S. aureus* plus dysuria, urinary frequency, or radiologic evidence of pyelonephritis.
- Other: radiologic evidence of infection plus a tissue culture positive for *S. aureus* in a body site.
- Unknown: does not fit the definition of other sites of infection.

We used descriptive statistics to characterize the population. Chi-square, Wilcoxon rank-sum, and multivariate logistic regression were used to determine the relationship between the primary outcome and the variables of interest. Factors with a univariate p-value < 0.3 were considered for the multivariate model. We performed all statistics using Statistical Analysis System version 9.0 (Cary, North Carolina). This study was approved by the Colorado Multiple Institutions Institutional Review Board.

## RESULTS

During the study period, we identified 178 patients with SAB of whom 43 were excluded: 39 (22%) had a hospital-onset infection; three patients had a second case of SAB during the study period; and one SAB was not speciated. Thus, 135 patients with CO-SAB were included. The median patient age was 55.7 years (interquartile range [IQR] 48.9–63.6), and 77% (n=105) were male. The most common comorbid conditions included diabetes mellitus (n=68, 50%), chronic kidney disease (n=28, 20%), IDU (n=27, 20%), cirrhosis (n=27, 20%), and malignancy (n=15, 11%). Twenty patients (15%) had a history of MRSA infection or colonization. The median Elixhauser score was 4.0 (IQR 3.0–5.0).

Skin and soft tissue infections were responsible for the largest proportion of SAB cases (n=65, 48%), followed by unknown source (n=38, 28%) and vascular catheters (n=19, 14%). MRSA bacteremia accounted for 32% (n=43) of CO-SAB cases. In comparison to those with MSSA bacteremia, patients with MRSA bacteremia were younger (odds ratio [OR] 0.5 for 10-year increments, 95% confidence interval [CI], 0.4–0.7) with higher Elixhauser comorbidity scores (OR 1.4 for one-unit increments; 95% CI, 1.1–1.7). Additionally, these patients were more likely to have a history of MRSA infection or colonization (OR 8.9; 95% CI, 2.7–29.7) and IDU (OR 2.4; 95% CI, 1.0–5.7) (Table).

## DISCUSSION

In this cohort of patients with CO-SAB, we found that patients with CO-MRSA bacteremia were younger, more likely to have previous MRSA colonization or infection, and more likely to have comorbid medical conditions than those with CO-MSSA bacteremia. We were surprised to see that both younger patients and those with comorbid conditions were at risk for CO-MRSA bacteremia. We suspect that this is due to an interaction between age and IDU (p = 0.003 on univariate analysis). Younger age was a previously described risk factor for CO-MRSA due to the USA300 strain, while increased comorbid conditions is a traditional risk factor for CO-MRSA. It would be interesting to determine if there is one predominant strain of MRSA in CO-MRSA at the current time, or if there are different strains prevalent in these two demographic groups.

Previous MRSA colonization or infection was the strongest risk factor for CO-SAB due to MRSA in our study. Our results are concordant with the work of Butler-Laporte et al.^13^ who found that the presence of a positive MRSA nares screen at any time in the past was associated with a high risk of MRSA infection in the context of a presumed SAB. Similarly, Bradley et al.^14^ reported that 12% of patients who newly acquire MRSA are hospitalized with an MRSA infection in the subsequent 18 months. Our study also correlates with the results of Yasmin et al.^4^ who reported that the majority of CO-MRSA bacteremia cases were due to skin and soft tissue infections. Yasmin et al.^4^ also found that having a central line within the previous 30 days was an independent risk factor with a calculated OR of 80 (95% CI, 2–3014). In our study, episodes of vascular access, including central line, dialysis catheters, peripheral IV, and venipuncture, were common sources of CO-SAB.

The changing epidemiology of MRSA is of interest to emergency care providers. As the first providers to evaluate patients with community-onset infections, emergency physicians are responsible for initiating appropriate antibiotic therapy both for resistant and susceptible pathogens. There are certainly risks to providing an insufficient spectrum of antibiotics to patients in the setting of sepsis. Patients who do not receive an appropriate antibiotic within the first three hours of presenting to the ED have increased mortality as compared to those who receive antibiotics that are active against the causative pathogen.^15^

However, there are also risks associated with administering antibiotics that are too broad in spectrum, including placing patients at higher risk for *Clostridioides difficile* colitis, encouraging the emergence of multidrug resistant organisms, and suboptimally treating severe infections. While most antibiotics incur some risk for *C. difficile* colitis, broad spectrum antibiotics such as clindamycin, third-generation cephalosporins, and fluoroquinolones place a patient at the highest risk for this infection.^16^ Additionally, the widespread use of an antibiotic can drive resistance in this organism within a hospital community. For example, Kim et al.^17^ described a relationship between increasing numbers of vancomycin doses administered at their hospital and an increasing number of patients with vancomycin-resistant enterococcus infection. Finally, broad spectrum therapy is not always the most effective antibiotic for a particular pathogen. Vancomycin is the most common empiric treatment of suspected MRSA bacteremia, but vancomycin is associated with inferior outcomes for MSSA bacteremia as compared to cefazolin or nafcillin therapy.^18^ By understanding risk factors for both MRSA and for MSSA, emergency physicians may be able to select antibiotics in a more nuanced fashion, choosing not only an adequate drug for *S. aureus* infection, but also the most effective therapy for either MRSA or MSSA based on the patient’s risk factors for the organism.

Emergency care providers also have the opportunity to promptly initiate appropriate infection control measures. If a patient has risk factors for MRSA, contact precautions may be implemented while the patient is still in the ED, appropriate environmental cleaning processes may be started, and a private inpatient room can be requested to decrease the risk of transmission to other vulnerable patients.

## LIMITATIONS

This study is limited by its single-center design. Results may not be applicable to facilities that care for a large population of outpatients with indwelling central lines or whose communities have different rates of MRSA. Further studies to characterize the strains of MRSA causing these infections would be an interesting correlate.

## CONCLUSION

CO-SAB continues to be prevalent in our urban community, with CO-MRSA accounting for almost one-third of SAB cases. Previous MRSA colonization was the strongest risk factor for current MRSA infection in this cohort of patients with CO-SAB. The demographics of adults with CO-MRSA bacteremia continue to evolve, currently being a hybrid between chronically-ill patients and those with young age. Emergency physicians must be aware of the changing risk factors for MRSA so that optimal antibiotic therapy and infection control measures can be initiated in a timely manner.

## Figures and Tables

**Table:** Demographic information plus univariate and multivariate odds ratios of risk factors for patients with community-onset methicillin-resistant and methicillin-susceptible *S. aureus* bacteremia.

	Population		Univariate		Multivariate		
				
Variable	MSSA N = 92	MRSA N = 43	OR	95% CI	OR	95% CI	P value
Age, median years (IQR)	58 (52–65)	52 (43–57)	0.95	0.92–0.98	0.5	0.4–0.7	< 0.0001
Elixhauser score, median (IQR)	4 (3–5)	5 (3–6)	1.18	0.98–1.41	1.4	1.1–1.7	0.01
Diabetes mellitus, n (%)	47 (51)	19 (44)	0.76	0.37–1.60			
Hemoglobin A1C, median (IQR)	9 (7–10)	9 (7–12)	1.11	0.90–1.37			
Chronic kidney disease, n (%)	21 (23)	7 (16)	0.65	0.25–1.67			
Hemodialysis, n (%)	9 (10)	3 (7)	1.45	0.37–5.63			
End-stage liver disease	18 (20)	7 (16)	0.79	0.30–2.06	0.4	0.1–1.3	0.13
Rheumatologic disease, n (%)	1 (1)	1 (2)	2.14	0.13–35.09			
Malignancy, n (%)	11 (12)	5 (12)	0.96	0.31–2.95			
Injection drug use, n (%)	14 (15)	13 (30)	2.38	1.00–5.66			
Human immunodeficiency virus, n (%)	1 (1)	2 (5)	4.39	0.39–49.80			
Intravascular device, n (%)	10 (11)	0 (0)					
History of MRSA infection or colonization, n (%)	5 (5)	14 (33)	8.4	2.78–25.34	8.9	2.7–29.7	< 0.0001
History of *S. aureus* bacteremia	5 (5)	1 (2)	0.41	0.05–3.66			
Presence of orthopedic hardware	6 (7)	5 (12)	1.89	0.54–6.56	3.2	0.8–12.8	0.10
Presumed source of infection							
Skin and soft tissue infection	28 (30)	15 (34)	1.22	0.57–2.64			
Vascular access	13 (14)	6 (14)	0.99	0.35–2.80			
Bone or joint infection	8 (9)	6 (14)	1.38	0.42–4.50			
Injection drug use	6 (6)	5 (11)	1.89	0.54–6.56			
Pneumonia	5 (5)	2 (5)	0.85	0.16–4.56			
Urinary tract infection	4 (4)	3 (7)	1.65	0.35–7.72			
Other	4 (4)	2 (5)	1.07	0.19–6.10			
Unknown source	25 (27)	5 (11)	0.37	0.13–1.06			

*MRSA*, methicillin-resistant *S. aureus*; *MSSA*, methicillin-susceptible *S. aureus*; *IQR*, interquartile range; *OR*, odds ratio;

*CI*, confidence interval.
